# Effective diagnosis of Alzheimer’s disease by means of large margin-based methodology

**DOI:** 10.1186/1472-6947-12-79

**Published:** 2012-07-31

**Authors:** Rosa Chaves, Javier Ramírez, Juan M Górriz, Ignacio A Illán, Manuel Gómez-Río, Cristobal Carnero

**Affiliations:** 1 Department of Signal Theory, Networking and Communications, University of Granada, c/Periodista Daniel Saucedo Aranda s/n, 18071, Granada, Spain; 2 Department of Nuclear Medicine, University Hospital Virgen de las Nieves, Granada, Spain; 3 Department of Neurology, University Hospital Virgen de las Nieves, Granada, Spain

## Abstract

**Background:**

Functional brain images such as Single-Photon Emission Computed Tomography (SPECT) and Positron Emission Tomography (PET) have been widely used to guide the clinicians in the Alzheimer’s Disease (AD) diagnosis. However, the subjectivity involved in their evaluation has favoured the development of Computer Aided Diagnosis (CAD) Systems.

**Methods:**

It is proposed a novel combination of feature extraction techniques to improve the diagnosis of AD. Firstly, Regions of Interest (ROIs) are selected by means of a *t*-test carried out on 3D Normalised Mean Square Error (NMSE) features restricted to be located within a predefined brain activation mask. In order to address the small sample-size problem, the dimension of the feature space was further reduced by: Large Margin Nearest Neighbours using a rectangular matrix (LMNN-RECT), Principal Component Analysis (PCA) or Partial Least Squares (PLS) (the two latter also analysed with a LMNN transformation). Regarding the classifiers, kernel Support Vector Machines (SVMs) and LMNN using Euclidean, Mahalanobis and Energy-based metrics were compared.

**Results:**

Several experiments were conducted in order to evaluate the proposed LMNN-based feature extraction algorithms and its benefits as: *i*) linear transformation of the PLS or PCA reduced data, *ii*) feature reduction technique, and *iii*) classifier (with Euclidean, Mahalanobis or Energy-based methodology). The system was evaluated by means of *k*-fold cross-validation yielding accuracy, sensitivity and specificity values of 92.78%, 91.07% and 95.12% (for SPECT) and 90.67%, 88% and 93.33% (for PET), respectively, when a NMSE-PLS-LMNN feature extraction method was used in combination with a SVM classifier, thus outperforming recently reported baseline methods.

**Conclusions:**

All the proposed methods turned out to be a valid solution for the presented problem. One of the advances is the robustness of the LMNN algorithm that not only provides higher separation rate between the classes but it also makes (in combination with NMSE and PLS) this rate variation more stable. In addition, their generalization ability is another advance since several experiments were performed on two image modalities (SPECT and PET).

## Background

### Alzheimer’s Disease (AD)

Alzheimer’s Disease (AD) is the most common cause of dementia in the elderly and affects approximately 30 million individuals worldwide [1]. Its prevalence is expected to triple over the next 50 years due to the growth of the older population. To date there is no single test that can predict whether a particular person will develop the disease. With the advent of several effective treatments of AD symptoms, current consensus statements have emphasized the need for early recognition [2].

### Functional brain imaging

Single Positron Emission Computed Tomography (SPECT) is a widely used technique to study the functional properties of the brain [3]. After the reconstruction and a proper normalization of the SPECT raw data, taken with Tc-99m ethyl cysteinate dimer (ECD) as a tracer, one obtains an activation map displaying the local intensity of the regional cerebral blood flow (rCBF). Therefore, this technique is particularly applicable for the diagnosis of neuro-degenerative diseases like AD [4,5]. On the other hand, Positron Emission Tomography (PET) measures the rate of glucose metabolism with the tracer [^18^*F*] Fluorodeoxyglucose. In AD, characteristic brain regions show decreased glucose metabolism, specifically bilaterally regions in the temporal and parietal lobes, posterior cingulate gyri and precunei, as well as frontal cortex and whole brain in more severely affected patients [6]. SPECT modality has lower resolution and higher variability than PET, but the use of SPECT tracers [7] is relatively cheap, and the longer half-lives when compared to PET tracers makes SPECT well suited, if not required, when biologically active radiopharmaceuticals have slow kinetics.

### Computer Aided Diagnosis (CAD)

In order to improve the prediction accuracy especially in the early stage of the disease, when the patient could benefit most from drugs and treatments, computer aided diagnosis (CAD) tools are desirable [8].

Several approaches for designing CAD systems of the AD can be found in the literature [9]. Univariate methodology is based on the analysis of regions of interest (ROIs) by means of some discriminant functions, whereas the second approach (multivariate) is related to statistical analysis techniques. Regarding the first, the most common and used approach is named Statistical Parametric Mapping (SPM) [10] software tool and its numerous variants. It was not developed specifically to study a single image, but for comparing groups of images. Regarding multivariate techniques, it is remarkable MANCOVA, which considers as one observation all the voxels in a single scan and requires a higher number of available samples than the one of features. This fact reports the well-known *small sample size* problem that is very common in nuclear medicine studies since the number of images is limited. In this work context, with the clear goal to solve the dimensionality issue, some techniques of feature space reduction were used and combined.

Firstly, a 3D binary mask is obtained from the average of control subjects which contains a set of activated voxels in certain brain regions characterized by an intensity level above half of the maximum intensity of the mean image. The use of activation masks and the automatic selection of spatial image components reports improved discrimination ability and reduces the complexity of the direct voxel as feature (VAF) approach [6]. The system was developed by exploring the masked brain volume in order to identify discriminant ROIs using different shaped subsets of voxels or components.

ROIs are defined as blocks of voxels represented by the so called Normalized Mean Square Error (NMSE) (further explanation in section Feature extraction) and are selected by means of a *t*-test [11]. These ROIs act as inputs for obtaining kernel Principal Component Analysis (KPCA), Partial Least Squares (PLS) or Large Margin Nearest Neighbours using a rectangular matrix (LMNN-RECT) in order to reduce the dimension of the feature vector to address the small sample size problem. In addition, it can be transformed the PLS or PCA space using a linear transformation matrix (denoted by **L**) that is built through the Euclidean distance based on the LMNN method that learns a linear transformation which attempts to make input neighbours share the same labels. This is achieved by minimizing a loss function (see section Loss function).

Finally, the classification task of the supervised learner is to predict by using several paradigms the class of an unknown pattern after a training procedure based on a subset of samples.

On the one hand, Support Vector Machines (SVMs) have achieved general success in the last decade [12-14] in the learning from examples paradigm and it can be considered as a special kind of large margin classifier. Recent developments in the definition and training of statistical classifiers make it possible to build reliable classifiers in very small sample size problems since SVM circumvents the curse of dimensionality, and even may find nonlinear decision boundaries for small training sets. On the other hand, LMNN classifier [15,16] aims to improve the Euclidean distance metric (which learns a linear transformation **L**, see section Large Margin Nearest Neighbors (LMNN)) by a new Mahalanobis one (which is described by the matrix **M **=** L **·**L**^*T*^, see also section Large Margin Nearest Neighbors (LMNN)) through linear transformations. In addition, Energy-based method is also analysed for LMNN, leading to further improvements in test error rates over the ones obtained with Euclidean or Mahalanobis distances as shown in Results and discussion Section. These transformations can improve significantly [17] in *k* Nearest Neighbors (KNN) [15] which are aimed to be organised to the same class, while examples from different classes are separated by a large margin [18,19].

## Methods

### Subjects and preprocessing

#### SPECT database

Baseline SPECT data from 97 participants were collected from the Virgen de las Nieves hospital in Granada (Spain). The patients were injected with a gamma emitting ^99*m*^Tc-ECD radiopharmeceutical and the SPECT raw data was acquired by a three head gamma camera Picker Prism 3000. A total of 180 projections were taken with a 2-degree angular resolution. The images of the brain cross sections were reconstructed from the projection data using the filtered backprojection (FBP) algorithm in combination with a Butterworth noise removal filter. The SPECT images are first spatially normalized using the SPM software, in order to ensure that voxels in different images refer to the same anatomical positions in the brain allowing us to compare the voxel intensities of different subjects. In this work, the images have been normalized using a general affine model, with 12 parameters ( [20-22]). After the affine normalization, the resulting image is registered using a more complex non-rigid spatial transformation model [21]. The deformations are parameterized by a linear combination of the lowest frequency components of the three-dimensional cosine transform bases [23]. A small-deformation approach is used, and regularization is by the bending energy of the displacement field. Then, we normalize the intensities of the SPECT images with respect to the maximum intensity, which is computed for each image individually by averaging over 3% of the highest voxel intensities, similarly as in [24]. After the spatial normalization, one obtains a 95 × 69 × 79 voxel representation of each subject, where each voxel represents a brain volume of 2 × 2 × 2m*m*^3^. The database is built up of imaging studies of subjects following the protocol of an hospital-based service. First, the neurologist evaluated the cognitive function, and those patients with findings of memory loss or dementia were referred to the nuclear medicine department in the *Virgen de las Nieves* hospital (Granada, Spain), in order to acquire complementary screening information for diagnosis^b^. Experienced physicians evaluated the images visually. The images were assessed using 4 different labels: Control (CTRL) for subjects without scintigraphic abnormalities and mild perfusion deficit (AD1), moderate deficit (AD2) and severe deficit (AD3), to distinguish between different levels of presence of hypo-perfusion patterns compatible with AD. In total, the database consists of *N *= 97 subjects: 41 CTRL, 30 AD1, 22 AD2 and 4 AD3 (see Table 1(a) for demographic details). Since the patients are not pathologically confirmed, the subject’s labels possess some degree of uncertainty, as the pattern of hypo-perfusion may not reflect the underlying pathology of AD, nor the different classification of scans necessarily reflect the severity of the patients symptoms. However, when pathological information is available, visual assessments by experts have been shown to be very sensitive and specific labeling methods, in contrast to neuropsychological tests [25,26]. Given that this is an inherent limitation of ’in vivo’ studies, our working-assumption is that the labels are true, considering the subject label positive when belonging to any of the AD classes, and negative otherwise. This work does not imply any experimental intervention and has been performed under the approval and supervision of the Clinical and Investigation Ethical Commission of the University Hospital Virgen de las Nieves (CEIC).

**Table 1:** Demographic details of the SPECT dataset and PET dataset

**(a)Demographic details of the SPECT dataset**			
	Num. of Samples	Sex (M/F) (%)	Age *μ* [range/*σ*]
CTRL	41	32.95/12.19	71.51 [46-85/7.99]
AD 1	30	10.97/18.29	65.20 [23-81/13.36]
AD 2	22	13.41/9.76	65.73 [46-86/8.25]
AD 3	4	0/2.43	76 [69-83/9.90]
**(b)Demographic details of the PET dataset**			
	Num. of Samples	Sex (M/F) (%)	Age *μ* [range/*σ*]
CTRL	75	29.33/20.67	75.97 [62-86/4.91]
AD	75	31.33/18.67	75.72 [55-88/7.40]

AD 1 = mild perfusion deficit, AD 2 = moderate deficit, AD 3 = severe deficit. *μ *and *σ *stands for population mean and standard deviation respectively.

#### PET database

PET data was obtained from the ADNI^a^ Laboratory on NeuroImaging (LONI, University of California, Los Angeles) website ( http://www.loni.ucla.edu/ADNI/). The ADNI was launched in 2003 by the National Institute on Aging (NIA), the National Institute of Biomedical Imaging and Bioengineering (NIBIB), the Food and Drug Administration (FDA), private pharmaceutical companies and non-profit organizations, as a 60 million, 5-year public- private partnership. The primary goal of ADNI has been to test whether serial magnetic resonance imaging (MRI), PET, other biological markers, and clinical and neuropsychological assessment can be combined to measure the progression of mild cognitive impairment (MCI) and early AD. Determination of sensitive and specific markers of very early AD progression is intended to aid researchers and clinicians to develop new treatments and monitor their effectiveness, as well as lessen the time and cost of clinical trials. The Principal Investigator of this initiative is Michael W. Weiner, MD, VA Medical Center and University of California – San Francisco. ADNI is the result of efforts of many co- investigators from a broad range of academic institutions and private corporations, and subjects have been recruited from over 50 sites across the U.S. and Canada. The initial goal of ADNI was to recruit 800 adults, ages 55 to 90, to participate in the research, approximately 200 cognitively normal older individuals to be followed for 3 years, 400 people with MCI to be followed for 3 years and 200 people with early AD to be followed for 2 years. For up-to-date information, see www.adni-info.org. FDG PET scans were acquired according to a standardized protocol. A 30-min dynamic emission scan, consisting of 6 5-min frames, was acquired starting 30 min after the intravenous injection of 5.0 ±0.5 mCi of ^18^F-FDG, as the subjects, who were instructed to fast for at least 4 h prior to the scan, lay quietly in a dimly lit room with their eyes open and minimal sensory stimulation. Data were corrected for radiation-attenuation and scatter using transmission scans from Ge-68 rotating rod sources and reconstructed using measured-attenuation correction and image reconstruction algorithms specified for each scanner. Following the scan, each image was reviewed for possible artifacts at the University of Michigan and all raw and processed study data was archived. Subsequently, the images were normalized through a general affine model, with 12 parameters [27] using the SPM5 software. After the affine normalization, the resulting image was registered using a more complex non-rigid spatial transformation model. The non-linear deformations to the Montreal Neurological Imaging (MNI) Template were parameterized by a linear combination of the lowest-frequency components of the three-dimensional cosine transform bases [28]. A small-deformation approach was used, and regularization was by the bending energy of the displacement field, ensuring that the voxels in different FDG-PET images refer to the same anatomical positions in the brains. After spatial normalization, an intensity normalization was required in order to perform direct images comparisons between different subjects. The intensity of the images was normalized to a value *I*_*max*_, obtained averaging the 0.1% of the highest voxel intensities exceeding a threshold. The threshold was fixed to the 10th bin intensity value of a 50-bins intensity histogram, for discarding most low intensity records from outside-brain regions, and preventing image saturation. Participant’s enrolment was conditioned to some eligibility criteria. General inclusion-exclusion criteria were as follows: 

• Normal control subjects: Mini Mental State Examination (MMSE) scores between 24−30 (inclusive), a Clinical Dementia Ratio (CDR) of 0, non depressed, non MCI, and non demented. The age range of normal subjects will be roughly matched to that of MCI and AD subjects. Therefore, there should be minimal enrolment of normals under the age of 70.

• Mild AD: MMSE scores between 20−26 (inclusive), CDR of 0.5 or 1.0, and meets NINCDS/ADRDA criteria for probable AD.

The PET database collected from ADNI consists of 150 labeled PET images: 75 control subjects and 75 AD patients (see Table 1(b) for demographic details). ADNI patient diagnostics are not pathologically confirmed, introducing some uncertainly on the subject’s labels. Using these labels, allows to test the robustness of the classifier. This should be also considered when comparing to other methods tested on autopsy confirmed AD patients, on which every classifier is expected to improve its performance [6].

Written informed consent was obtained from all ADNI participants before protocol-specific procedures were performed. The informed consent not only covers consent for the trial itself, but for the genetic research, biomarker studies, biological sample storage and imaging scans as well. The consent for storage includes consent to access stored data, biological samples, and imaging data for secondary analyses. By signing the consent, ADNI participants authorize the use of the data for large scale, multicenter studies that combine data from similar populations.

## Feature extraction

In this article, we propose to apply a combination of different extraction methods in order to obtain the most important features in the early diagnosis of AD. In this way, we can save the memory space and reduce the system complexity removing those useless and harmful noisy components. We are also able to deal with data set of few samples and high dimensions and thus weakening the disadvantages caused by the so-called curse-of-dimensionality problem [16].

**Figure 1:**
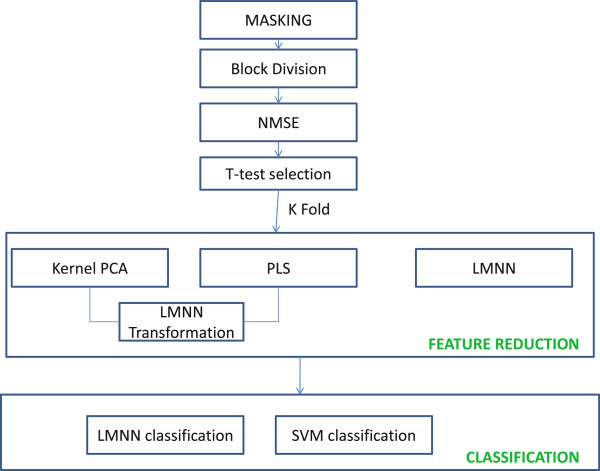
**Feature extraction and classification diagram.** Voxels and Features, combination of feature reduction techniques and classifiers evaluated with *k*-Fold cross validation.

As detailed in Figure 1, first of all the masking process is done. Control subjects are averaged in a tridimensional image ***sm(x,y,z)***. In functional imaging, each voxel carries a grey intensity level *I*(**x**_*j*_), which is related to the regional cerebral blood flow, glucose metabolism, etc. in the brain of a patient, depending on the image acquisition modality. Secondly, it is obtained a 3D ***mask(x,y,z)*** that consists of all the voxels with ***sm(x,y,z) ***>* a*_*T*_. The threshold *a*_*T*_ is equivalent to the 50% of the maximum intensity in ***sm(x,y,z)***.

Secondly the Block Division is done as shown in Figure 1. Baseline VAF is a way of including in ***vaf(x,y,z)*** all the voxels inside the obtained ***mask(x,y,z)*** and considering them as features. Therefore, voxels outside the brain and poorly activated regions are excluded from this analysis. The main problem to be faced up by these techniques is the well-known small sample size problem, that is, the number of available samples is much lower than the number of features used in the training step. However in this work, the combination of feature reduction techniques does not only solve this problem, but also helps to reach better results of classification.

Finally, instead of using directly all the voxels, the regions are considered in 3D because not all the brain regions provide the same discriminant value for detecting the early AD. In fact, the posterior cingulate gyri and precunei, as well as the temporo-parietal region are typically affected by hypo-perfusion in the AD [14]. That is the reason why, each functional image is processed by means of 3D *v *×* v *×* v* cubic voxels defining ROIs, or ***block(x,y,z)*** centered in *(x,y,z)* coordinates which belong to ***vaf(x,y,z)***. Then, it is calculated the Normalized Minimum Squared Error or *NMSE*_*p*_*(x,y,z)* defined as:

(1) $$\begin{array}{l} \text{NMS}E_{p}(x,y,z)\\ \;=\;\frac{\overset{v}{\underset{l,m,n=-v}{\sum }}{\left[f(x\;-\;l,y\;-\;m,z\;-\;n)\;-\;g_{p}(x\;-\;l,y\;-\;m,z\;-\;n)\right]}^{2}}{\overset{v}{\underset{l,m,n=-v}{\sum }}{\left[f(x\;-\;l,y\;-\;m,z\;-\;n)\right]}^{2}} \end{array}$$

It is obtained for each subject and block (see Figure 1) where *f*(*xyz*) is the mean voxel intensity of all the control subjects and *g*_*p*_(*xyz*) is the voxel intensity of the *p*-th subject at (*xyz*) coordinates. The most discriminant ROIs are obtained by means of an absolute value two-sample *t*-test with pooled covariance estimate on NMSE features as in [14].

Widely used methods for the analysis of data sets are PCA [29,30] and projections to latent structures (PLS) [31,32], that work computationally well for many variables and observations. By contrast, LMNN algorithm is aimed at the organization of the k-nearest neighbors to the same class, while examples from different classes are separated by a large margin [15,17,33,34].

In this work we propose and compare several feature extraction methods (shown in Figure 1) that includes on the one hand the combination of NMSE with PCA (see section Large Margin Nearest Neighbors (LMNN)) or PLS (see section Partial Least Squares (PLS)) plus the LMNN transformation. On the other hand, NMSE is directly combined with a LMNN-RECT reduction (see section LMNN-RECT as feature reduction technique).

### Principal Component Analysis: PCA

PCA is a multivariate approach often used in neuroimaging to significantly reduce the original high-dimensional space of the brain images to a lower dimensional subspace [35]. PCA generates an orthonormal basis vector that maximizes the scatter of all the projected samples, which is equivalent to find the eigenvalues from the covariance matrix. PCA can be used in combination with the so-called kernel methods [36]. The basic idea of the kernel PCA [37] method (further details in appendix 1: Kernel PCA) is to first pre process the data by some non-linear mapping and then to apply the same linear PCA.

### Partial Least Squares (PLS)

PLS can model the observed variables **X **(representing the feature space of input) and **Y **(representing the labels) by means of linear latent variables (not directly observed or measured) according to the regression models [38,39]:

(2) $$X=T\cdot P^{T}+E_{x}$$

(3) $$Y=U\cdot Q^{T}+E_{y}$$

where **T**, **U** are the score matrices; **E**_**x**_, **E**_**y**_ are the error matrices and **P**, **Q** are the loading matrices with number of columns being the number of PLS components. The score matrices result from projection of the data matrices **X **and **Y **on loading matrices. The fundamental goal of PLS is to maximize the covariance between the scores of **X** and **Y**. PLS can be used as a regression tool or as a dimension reduction technique similar to PCA. The main difference between PLS and PCA is that the former creates orthogonal weight vectors by maximizing the covariance between the variables **X** and **Y**, thus, PLS does not only consider the variance of the samples but also the class label [40]. Partial least squares modeling [40] is an effective method for feature extraction that has shown improved results over other conventional feature extraction methods such as PCA in classification problems. In this work, PLS is implemented by means of SIMPLS algorithm (further details in Appendix 2: Partial Least Squares SIMPLS algorithm).

### Large Margin Nearest Neighbors (LMNN)

Distance metric [41] is a key issue in many machine learning algorithms. LMNN is used in this work in different ways: *i*) as a transformation of the feature space obtained by means of PLS or PCA in order to better separate the control subject and AD patient classes, *ii*) as feature reduction technique by performing the transformation as a rectangular matrix (LMNN-RECT), and *iii*) as a classifier as reported in section Large margin nearest classifier.

The objective of LMNN is to obtain a family of metrics over the feature space. Let {(**x**_*i*_,**y**_*i*_)} denote a training set of *n* labeled examples with inputs $x_{i}\varepsilon R^{d}$ and associated class labels **y**_**i**_. Our goal is to learn a linear transformation **L**: $R^{d}\to R^{d}$. These metrics compute squared distances as:

(4) $$D_{L}(x_{i},x_{j})={∥L(x_{i}-x_{j})∥}_{2}^{2}$$

Equation 4 is commonly used to express squared distances in terms of the squared matrix:

(5) $$M=L^{T}\cdot L$$

On the other hand, the squared distances are denoted as Mahalanobis metrics in terms of **M**:

(6) $$D_{M}(x_{i},x_{j})={\left(x_{i}-x_{j}\right)}^{T}\cdot M\cdot (x_{i}-x_{j})$$

A Mahalanobis distance can be parameterized in terms of the matrix **L** or the matrix **M**[15]. The first is unconstrained, whereas the second must be positive semidefinite.

The main idea of LMNN consists of minimizing the loss function (see the following section Loss function) that is able to learn a distance metric under which inputs and their target neighbours are closer together.

#### Loss function

In LMNN, target neighbours are defined as input patterns of the same class that are wanted to be closer. The loss function to be minimized consists of two terms. One acts to *pull* target neighbours closer together penalizing large distances between each input and its target neighbours. The other term acts to *push* differently labeled examples further apart. It penalizes small distances between differently labeled examples. The *pull* term is represented by the following equation:

(7) $$\varepsilon_{\text{pull}}(L)=\underset{j\to i}{\sum }{∥L(x_{i}-x_{j})∥}^{2}$$

where *j *→* i* means that input **x**_**j**_ is a target neighbour of input **x**_**i**_. A new indicator variable is introduced to define the *push* term of the loss function:

(8) $$y_{\mathrm{il}}=\left\{\begin{array}{c} 1\;\text{if}\;y_{i}=y_{l}\\ 0\;\text{otherwise} \end{array}\right)$$

so that:

(9) $$\begin{array}{c} \varepsilon_{\text{push}}(L)=\underset{i,j\to i}{\sum }\underset{l}{\sum }(1-y_{\mathrm{il}})\cdot {\left[1+{∥L(x_{i}-x_{j})∥}^{2}-{∥L(x_{i}-x_{l})∥}^{2}\right]}_{+} \end{array}$$

where []_+_ =* max*(*z*,0) denotes the standard hinge loss [15].

Finally, we combine the two terms *ε*_*pull*_(**L**) and *ε*_*push*_(**L**) into a single loss function for distance metric learning. The two terms can have competing effects, to attract target neighbours and to repel impostors. Impostors are defined as the inputs with different labels. A weighting parameter *μ *∈[0,1] balances these goals.

(10) $$\varepsilon (L)=(1-\mu )\cdot \varepsilon_{\text{pull}}(L)+\mu \cdot \varepsilon_{\text{push}}(L)$$

#### LMNN-RECT as feature reduction technique

The loss function needs to be optimized in order to obtain the distance metric transforms in terms of the explicitly low-rank linear and rectangular matrix transformation **L**. The optimization over **L** is not convex unlike the original optimization over **M**, but a (possibly local) minimum can be computed by standard gradient-based methods. We call this approach LMNN-RECT [42], in which **L** is a matrix with a size equal to the number of features selected by the *t*-test. In particular, in this work the matrix **L** is multiplied by the matrix consisting of the NMSE features selected by the *t*-test and defined above in order to obtain a new space of features that better separates control subjects from AD patients. This fact is experimentally demonstrated in the Results and discussion Section.

#### Kernel LMNN

It is interesting to consider the case where **x**_**i **_are mapped into a high dimensional feature space *ϕ*(**x**_**i**_) and a Mahalanobis distance is sought in this space. We focus on the case where dot products in the feature space may be expressed via a kernel function, such that

(11) $$\phi (x_{i})\cdot \phi (x_{j})=k(x_{i},x_{j})$$

for some kernel k [19]. When we use the Kernel PCA trick framework (appendix 1), the original LMNN can be immediately used as Kernel LMNN (KLMNN) as it is explained in [43]. The new KPCA trick framework offers several practical advantages over the classical kernel trick framework, e.g. no mathematical formulas and no reprogramming are required for a kernel implementation, a way to speed up an algorithm is provided with no extra work, the framework avoids troublesome problems such as singularity.

### Feature/model selection

**Figure 2:**
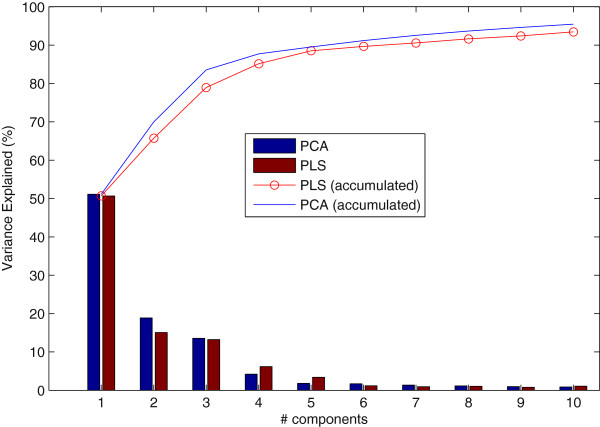
**Feature/Model Selection by means of Variance Explained.** Variance Explained (%) versus PCA and PLS Components in bar diagram. Lines represent the accumulated Variance Explained (%) versus Principal Components and PLS Components.

The number of features used is a trade-off between ROIs that are really important and that do not worsen the computational time of the CAD. We demonstrated experimentally that 200 NMSE features (ROIs) is a number high enough to guarantee the quality of the image in posterior classification. However, this number must be reduced in order to improve the computational time of the system with strategies such as PCA, PLS or LMNN-RECT. The final number of features used has been experimentally tuned by the observation of Figure 2 in which the percentage of variance explained for features (PCA or PLS) chosen are drawn as bars and a line represents the cumulative Variance Explained. In the case, Variance Explained accounts for the variation of a feature subset when PCA or PLS strategies are applied. In this graphic, we can observe that up to six components, the variance explained for PCA and PLS does not change significantly.

## Classification

LMNN and SVM classifiers were used in this work to build the AD CAD system. They present many similarities, for example its potential to work in nonlinear feature spaces by using the kernel trick. On the other hand, features can be extracted by means of the kernel trick and PCA (kernel PCA, KPCA) or LMNN (kernel LMNN, KLMNN) [43]. LMNN can be viewed as the logical counterpart to SVMs in which kNN classification replaces linear classification. However, LMNN contrasts with classification by SVMs, in that it requires no modification for multiclass problems that involve combining the results of many binary classifiers, that is there is no explicit dependence in the number of classes.

### Large margin nearest classifier

Some techniques were developed to learn feature weights to manage the change of distance structure of samples in nearest neighbour classification. Euclidean distance, the most commonly used, assumes that each feature is equally important and independent from others. By contrast, a distance metric with good quality such as Mahalanobis, should identify relevant features assigning different weights or importance factors to the extracted ROIs [44]. Only when the features are uncorrelated, the distance under a Mahalanobis distance metric is identical to that under the Euclidean distance metric. On the other hand, our work has been inspired by energy-based metric (EBC) learning, obtaining with it the best results in terms of accuracy, specificity and sensitivity [33,45]. EBC consists of computing the loss function for every possible label **y**_**i**_. We compute the minimization of three terms. The first one term is defined to be the squared distances to the *k* target neighbours of **x**_**i**_. The second term accumulates the hinge loss over all impostors (that is differently labeled) which invade the perimeter around **x**_**i**_ determined by its target neighbours. The third term is the accumulation of the hinge loss for differently labelled examples whose perimeters are invaded by **x**_**i**_.

### Support vector machines classifier

SVMs [46,47] let to build reliable classifiers in very small sample size problems [48] and even may find nonlinear decision boundaries for small training sets. SVM [13] separates a set of binary-labeled training data by means of a maximal margin hyperplane, building a decision function $R^{N}\to \left\{\pm 1\right\}$. The objective is to build a decision function **f**: $R^{N}\to \left\{\pm 1\right\}$ using training data that is, *lN*-dimensional patterns **x**_**i **_and class labels *y*_*i*_: (**x**_1_*y*_1_), (**x**_2_*y*_2_), …, (**x**_*l*_*y*_*l*_), so that f will correctly classify new unseen examples (**x***y*). Linear discriminant functions define decision hyperplanes in a multidimensional feature space: **g**(***x***) =*** w***^*T *^·** x** + *w*_0_ where w is the weight vector to be optimized that is orthogonal to the decision hyperplane and *w*_0_ is the threshold. The optimization task consists of finding the unknown parameters *w*_*i*_, *i *= 1, …, *N* and *w*_0_ that define the decision hyperplane. When no linear separation of the training data is possible, SVM can work effectively in combination with kernel techniques such as quadratic, polynomial or radial basis function (RBF), so that the hyperplane defining the SVM corresponds to a non-linear decision boundary in the input space [14].

## Results and discussion

Several experiments were conducted in order to evaluate the proposed LMNN-based feature extraction algorithms and its benefits as: *i*) linear transformation of the PLS or PCA reduced data, *ii*) feature reduction technique, and *iii*) classifier (with Euclidean, Mahalanobis or Energy-based methodology). SVM classification including transformation of the input space by means of linear, polynomial, quadratic or rbf kernels, which define non-linear decision surfaces, was adopted for the first two approaches. The classification performance of our approach was tested by means of *k*-fold cross validation (instead of Leave-One-Out), which is widely used to compare the performances of different predictive modelling procedures as in [49].

Although there are studies that consider *k* independent training and test splits (for instance in [50,51]), we focus on the standard *k*-fold cross-validation that is widely used ( [6,51,52]). In *k*-fold procedure, there is no overlap between test sets: each example of the original data set is used once and only once as a test example. In *k*-fold cross-validation, sometimes called rotation estimation, the dataset *D* is randomly split into k mutually exclusive subsets (the folds) *D*_1_, *D*_2_,…,*D*_*k *_of approximately equal size. The inducer is trained and tested k times; each time t *ε*{*t*_1_*t*_2_,…,*t*_*N*_}, it is trained on D-{*D*_*t*_} and tested on *D*_*t*_[53]. 10 folds were used in each experiment which yielded accurate estimates of the error rates. For each iteration (t = 1,…,10), the algorithm returns randomly generated indices for a *k*-fold cross-validation of D observations. Testing rate is mostly equal to the integer of the fraction 100/number of folds, that is 10% in our experiments, but it can vary randomly one or two samples in each iteration if the number of observations is a prime number. These indices are used for testing and the rest (approximately 90%) for training. Statistical results obtained in each iteration are averaged.

Thus, by using cross-validation, several feature extraction and classification methods were objectively compared in terms of their respective fractions of misclassified samples. In this way, the classifier was evaluated in depth as a tool for the early detection of AD in terms of the accuracy (Acc), sensitivity (Sen) and specificity (Spe), which are defined as:

$$\begin{array}{c} \text{Sensitivity}=\frac{\text{TP}}{\text{TP}+\text{FN}};\;\text{Specificity}=\frac{\text{TN}}{\text{TN}+\text{FP}} \end{array}$$

respectively, where *TP* is the number of true positives: number of AD patients correctly classified; *TN* is the number of true negatives: number of control subjects correctly classified; *FP* is the number of false positives: number of control subjects classified as AD patients; *FN* is the number of false negatives: number of AD patients classified as control subjects.

For posterior analysis, the data was arranged in two different Groups: AD subjects were labeled as positive and controls as negative. The motivation of doing that is to test our method with all the available stages of the disease, keeping the database as balanced as possible (41 CTRL versus 56 AD for SPECT and 75 CTRL versus 75 AD for PET) and to include several types of patterns in the classification task (training and test).

In the feature reduction process, there are certain parameters to tune such as the number of NMSE-Blocks, the number of PCA, PLS or LMNN reduced features and the selection of the kernel shape (linear, polynomial, quadratic or RBF) which define better decision surfaces in SVM classification. The NMSE features were computed using 5 × 5 × 5 voxel blocks since reduced size cubic NMSE features yield better results as shown in [14]. Furthermore, 200 discriminant features were selected by means of *t*-test reduction (a higher number of NMSE blocks means a decrease of the classification method effectiveness). The posterior reduction of the size of the feature vector is achieved by means of PCA, PLS or LMNN-RECT.

### Experiments with SPECT database

**Figure 3:**
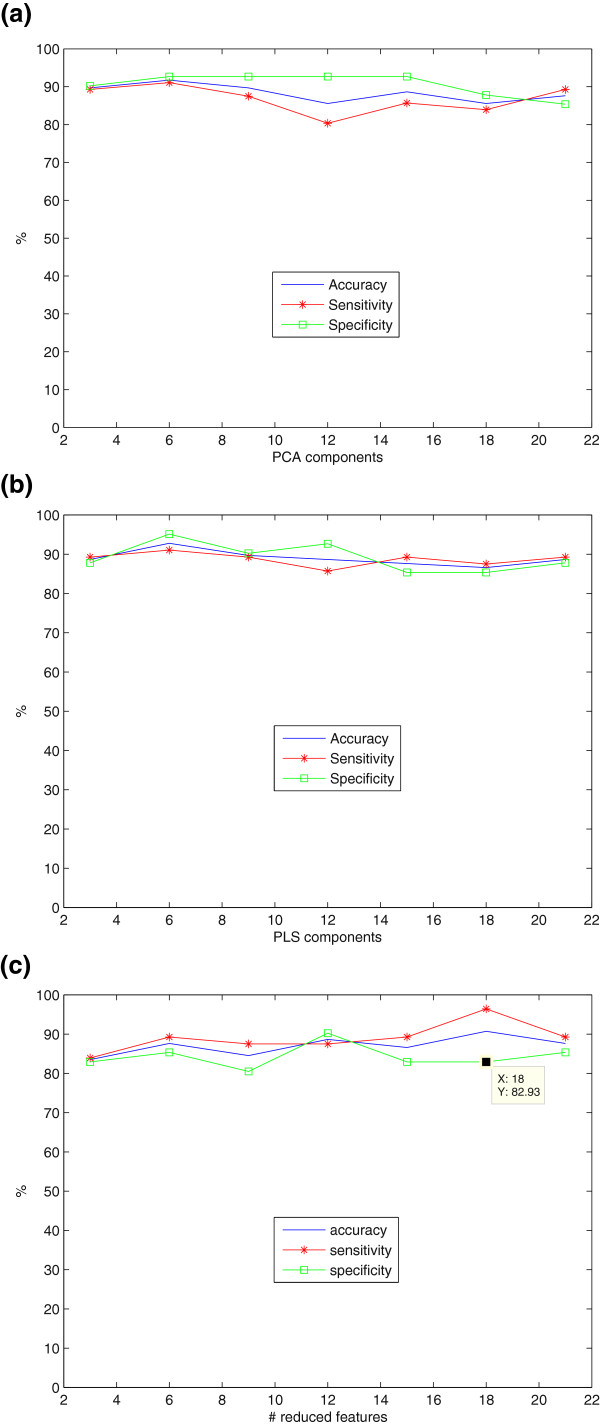
**SVM classification: Accuracy, Specificity and Sensitivity (%) versus number of reduced features for SPECT.** Feature reduction techniques: **a**) PCA, **b**)PLS, **c**)LMNN-RECT.

In a first experiment, the different feature extraction techniques considered in this work (PCA+LMNN, PLS+LMNN and LMNN-RECT) were compared in Figure 3(a)- 3(c). All the feature extraction methods were found to be very robust to the selection of the number of input features to the classifier. To conclude, when six features were used for classification, PCA-LMNN yielded Acc = 91.75%, Sen = 91.07% and Spe = 92.68%, while PLS-LMNN outperformed these values yielding Acc = 92.78%, Sen = 91.07% and Spe = 95.12%. As LMNN-RECT is concerned, the best results were obtained when 18 features are transformed: Acc = 90.72%, Sen = 96.43%, Spe = 82.93%. The advantage of the last method is its speed since it does not need the combination with another reduction technique nor space transformation LMNN.

**Figure 4:**
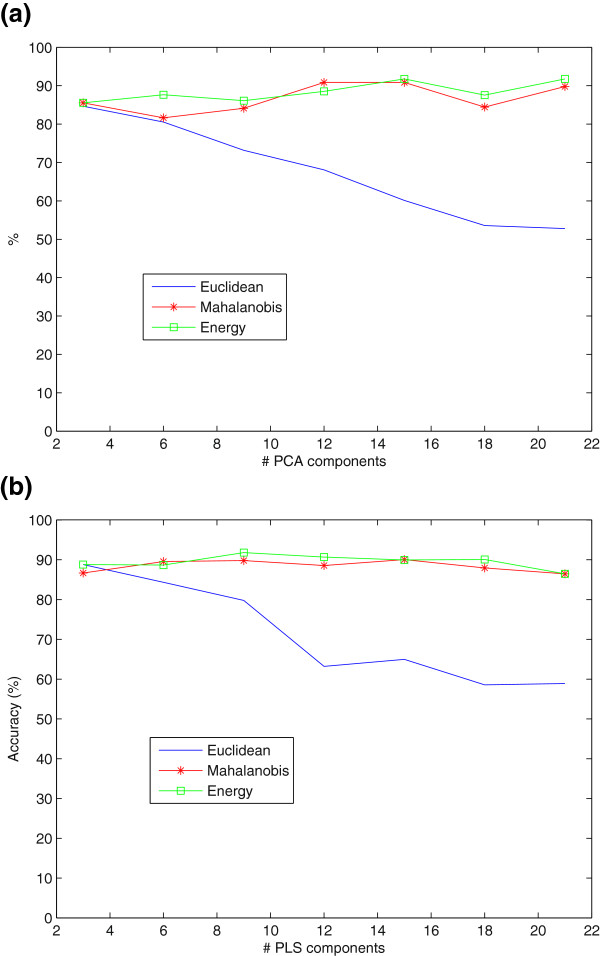
**LMNN classification (Euclidean, Mahalanobis and Energy-based models) for SPECT.** Feature reduction techniques: **a**)PCA, **b**)PLS.

The second experiment analyzes classification using LMNN using Euclidean, Mahalanobis and Energy-based models when NMSE-PCA or NMSE-PLS features are considered. Figures 4(a)and 4(b) show the accuracy obtained by LMNN classification as a function of the number of PCA coefficients and PLS coefficients, respectively. The results show that LMNN classification using energy-based models and Mahalanobis distances performs better than when the Euclidean distance is considered, which suffers a decrease in the accuracy as the number of features increases. LMNN classification using energy-based models and Mahalanobis distances were found to be very robust against the selection of the dimension of the feature vector yielding peak values of the accuracy of 91.76% and 90.87%, respectively, when NMSE-PCA features are used. If PLS technique is used instead of PCA, the accuracy results improved yielding accuracy values of 91.78% and 89.78% for energy-based models and Mahalanobis distance, respectively. In all these cases, energy-based models outperformed the others.

**Figure 5:**
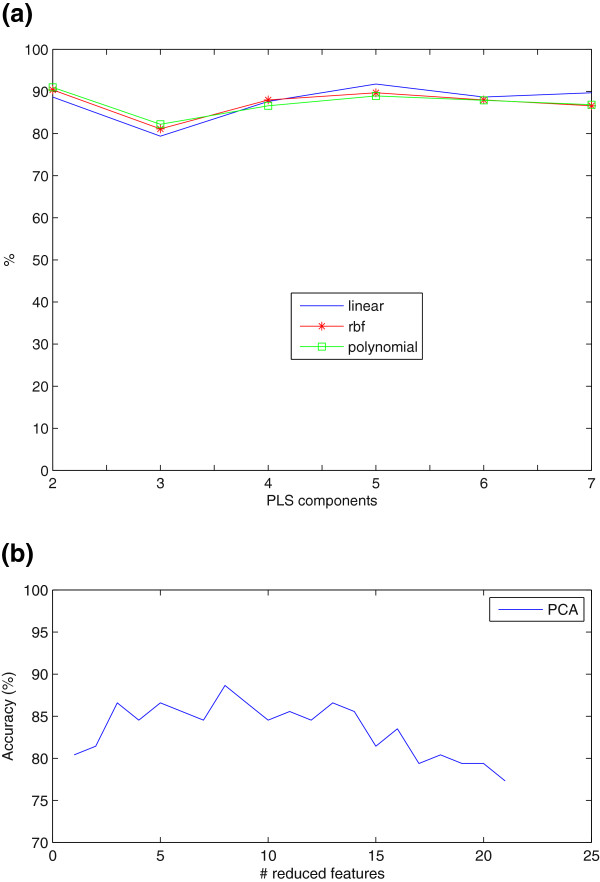
a) Kernel SVM for PLS features LMNN transformed b) linear SVM over PCA features directly (reduced to the half VAF) obtained for SPECT.

Since PLS feature extraction in combination with a LMNN transformation reported the best results and, aimed at further improving the accuracy of the classification, the selection of the best kernel-transformation of the input space by means of kernels and SVM was analyzed. Figure 5(a) shows the accuracy of the system as a function of the number of PLS coefficients for linear, polynomic and RBF kernel-based SVM classification. In conclusion, linear kernel outperformed the others with a 92.7% of accuracy.

It is remarkable the fact that when using the combination of 3-D NMSE blocks as input features and afterwards transformed them with LMNN algorithm in its multiple possibilities (both as reduction technique, linear transformer or classifier) adds a valuable robustness to the system. This can be proven in view of the experiments shown in Figures 3(a), 3(b), 3(c), 4(a), 4(b), 5(a). In Figure 5(b), PCA was used directly over the voxels reduced to the half (because of the high computational cost) and treated with the same type of mask as explained in this work. The results in Figure 5(b) showed that the variation of accuracy increases when voxels are used as features. By contrast, in this work the advantage of the combination of the methods proposed, is that they maintain stable around the 90%. We can conclude that the fact of obtaining the ROIs by using the combination of NMSE Blocks with LMNN algorithm favors the stability in all the range of reduced features, thus promoting the robustness of the algorithm.

Finally, Table 2 shows the accuracy, sensitivity and specificity rates of the proposed methods and compares them with other recently reported techniques including VAF, Gaussian Mixture Models (GMM) and PCA [36,54-56]. Based on the analysis shown in section Feature/model selection regarding the feature selection model, PCA and PLS feature extraction considered up to six features that retain most of the variance of the data. It can be concluded from the Table 2 that the LMNN transformation when combined with PCA or PLS yields the best results and reports benefits when compared to other reference methods.

**Table 2:** Statistical measures of performance of LMNN-based techniques in comparison with other reported methods for SPECT database

**SVM-linear classifier**	**Accuracy (%)**	**Sensitivity (%)**	**Specificity(%)**
VAF	83.51	83.93	82.93
PCA	86.56	91.07	80.49
GMM	89.69	90.24	89.29
**Gaussian kernel PCA+LMNN Transformation**	**91.75**	**91.07**	**92.68**
**Gaussian kernel PLS+LMNN Transformation**	**90.72**	**91.07**	**90.24**
**PLS+LMNN Transformation**	**92.78**	**91.07**	**95.12**
**LMNN-RECT**	**80.28**	**70**	**87.80**
**LMNN-Classifier Accuracy** (%)	**Euclidean**	**Mahalanobis**	**Energy**
**PCA**	**80.54**	**81.63**	**87.65**
**PLS**	**84.33**	**89.56**	**88.67**

SVM classifier: Comparison of the methods reported in this work with VAF, GMM and PCA operation points. LMNN-based techniques parameters: linear SVM classifier with 6 components. VAF parameters: linear SVM classifier, GMM parameters: *σ *= 6 RBF-SVM classifier with 8 components and PCA parameters: *σ *= 6 RBF-SVM classifier with 16 components. LMNN Classifier with 6 components: Euclidean, Mahalanobis and Energy.

To sum up, LMNN was presented as a valid solution to make broader the margin between the classes. It was developed an effective CAD system in which it is not necessary to incorporate an *a priori* knowledge about the pathology, since up to its first feature extraction step, all the voxels with a considerable activation (that is, those voxels that are located inside the calculated mask) are considered. The analysis shown in this papers reports clear advantages in the following ROI-selection steps as well, because they were computed in an automatic way for the early diagnosis of Alzheimer’s disease. The best combination of feature reduction techniques yielded an accuracy value of 92.72%, thus outperforming other recently and consolidated reported methods such as VAF, PCA and GMM (Table 2). Finally, in order to study in depth the AD classification with LMNN-based techniques , we have also included additional information about the classification of AD1 subjects versus CTRL. This set up is more difficult to be classified since AD1 pattern is still a challenge to be diagnosed. If we only consider the case CTRL versus AD1 the precision rates of the method are for PCA plus LMNN: Acc = 84.51%, Sen = 73.33%, Spe = 92.68%, for PLS plus LMNN transformation: Acc = 83.10%, Sen = 70%, Spe = 92.68% and for LMNN-RECT: Acc = 84.51%, Sen = 76.67%, Spe = 90.24%. These results still represent a great advance in the field in comparison with the baseline VAF: Acc = 77.46%, Sen = 70%, Spe = 82.93%.

### Experiments with PET database

**Figure 6:**
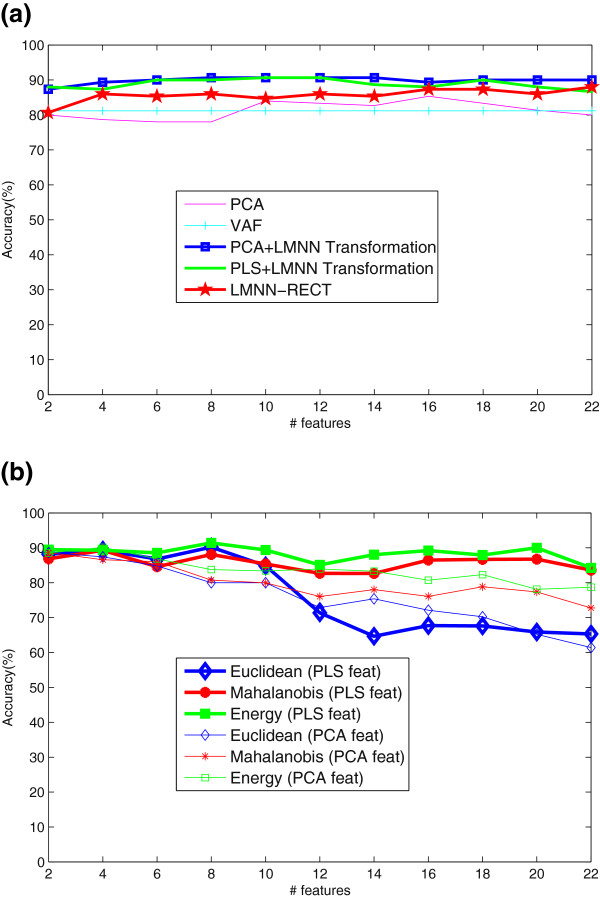
**a) SVM classification: Accuracy, Specificity and Sensitivity (%) versus number of reduced features for PET database.** Feature reduction techniques: PCA plus LMNN Transformation, PLS plus LMNN Transformation, LMNN-RECT, PCA and VAF **b)** LMNN classification (Euclidean, Mahalanobis and Energy-based models) for PCA and PLS features.

Additionally, several experiments were performed on a PET database in order to highlight the generalization ability of the proposed method. The same parameters such as voxel size or number of NMSE Blocks than for SPECT data were used. Figure 6(a)shows the different feature extraction techniques of this work, that is, PCA or PLS plus LMNN transformation and LMNN-RECT in comparison with PCA or VAF baseline when a linear SVM classification is performed. In the light of the graphic, this manuscript technique reaches a maximum accuracy rate of 90.67% (88% sensitivity and 93.33% specificity) for both PCA and PLS plus LMNN transformation and when used LMNN-RECT, accuracy 87.33% (82.67% sensitivity and 92% specificity), thus outperforming the PCA (Acc = 85.33%) or baseline VAF (Acc = 81.18%) techniques.

Figure 6(b) shows LMNN classification using energy-based models, Mahalanobis and Euclidean distances for PCA and PLS features. Maxima accuracy rates were obtained for Energy-based classifier (90.11% for PCA and 89.99% for PLS).

### ROC analysis

**Figure 7:**
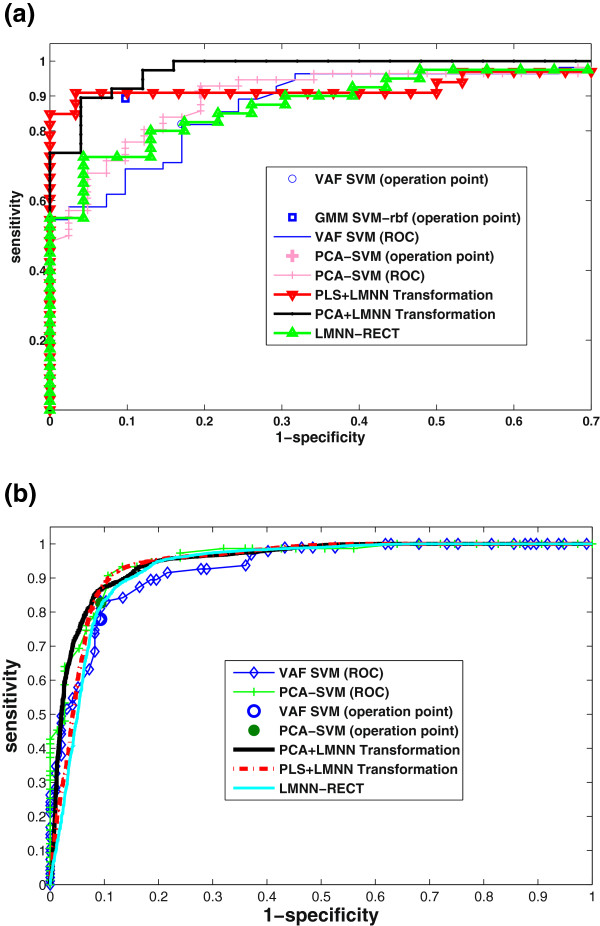
**ROC Analysis.** LMNN-based methods SVM classified (PCA+LMNN Transformation, PLS+LMNN Transformation, LMNN-RECT). Comparison to other recently reported methods represented by their operation points. a) SPECT database. The AUC obtained for each ROC is: PCA+LMNN (0.9411), PLS+LMNN (0.9424), LMNN-RECT (0.9076), VAF SVM (0.8993) and PCA SVM (0.9177). b)PET database. The AUC obtained for each ROC is: PCA+LMNN (0.9505), PLS+LMNN (0.9437), LMNN-RECT (0.9325), VAF SVM (0.8500) and PCA SVM (0.9006).

Figures 7(a) and 7(b) show the receiver operating characteristic (ROC) curves of the proposed systems and other methods that were considered as a reference for SPECT and PET databases respectively. Several experiments were carried out on the different image modalities (SPECT and PET) in order to highlight the generalization ability of the proposed method. The analysis shows that the presented CAD system based on LMNN algorithm and SVM yields the best trade-off between sensitivity and specificity by shifting the operating point up and to the left in the ROC space [57] in comparison with other reported methods such as VAF SVM, PCA SVM and GMM SVM. As shown in Figure 7(a), PLS plus LMNN transformation provides an operation point located in the upper left than other approaches on the ROC space. In addition, the improvement of the proposed LMNN-based technique is also supported by the AUC analysis for SPECT/PET databases respectively: PLS plus LMNN transformation: 0.9424/0.9437, PCA plus LMNN transformation 0.9411/0.9505, LMNN-RECT: 0.9076/0.9325 that outperform the AUC of other reported methods such as VAF SVM: 0.8993/0.8500 and PCA SVM: 0.9177/0.9006.

## Conclusions

Kernel Distance Metric Learning Methods were investigated for SVM-based classification of SPECT brain images in order to improve the early AD’s diagnosis. Several experiments were conducted in order to evaluate the proposed LMNN-based feature extraction algorithms and its benefits as: *i*) linear transformation of the PLS or PCA reduced data, *ii*) feature reduction technique, and *iii*) classifier (with Euclidean, Mahalanobis or Energy-based methodology). LMNN classification using energy-based models and Mahalanobis distances performs better than when the Euclidean distance is considered, which suffers a decrease in the accuracy as the number of features increases. Aiming at further improving the accuracy of the classification, SVM was also compared to LMNN-based classification yielding improved results. Thus, the proposed methods yielded Acc rates of 92.7% for SPECT and 90.11% for PET when an advanced feature extraction technique consisting of NMSE feature selection, PLS feature reduction and LMNN transformation in combination with linear SVM classification was considered, thus outperforming other recently and consolidated reported methods such as VAF, PCA or GMM. One of the principal advantages of our techniques is the robustness and stability of the proposed methods shown in this work as stated in the Results. Another property is its generalization ability in the light of the results obtained with an PET database.

## Endnotes

^a^ Data used in preparation of this article were obtained from the Alzheimer’s Disease Neuroimaging Initiative (ADNI) database (adni.loni.ucla.edu). As such, the investigators within the ADNI contributed to the design and implementation of ADNI and/or provided data but did not participate in analysis or writing of this report. A complete listing of ADNI investigators can be found at: http://adni.loni.ucla.edu/wp-content/uploads/how_to_apply/ADNI_Acknowledgement_List.pdf

^b^ Clinical information is unfortunately not available for privacy reasons, but only demographic information.

## Appendix

## Appendix 1: Kernel PCA

In kernel PCA, each vector **x** is projected from the input space, $R^{n}$, to a high dimensional feature space $R^{f}$ by a non-linear mapping function where *ϕ*: $R^{n}\to R^{f}$ with *f *>* n*. Note that the dimensionality of the feature space can be arbitrarily large [56]. In $R^{f}$, the corresponding eigenvalue problem is

(12) $$C^{\phi }\cdot \omega^{\phi }=\lambda \cdot \omega^{\phi }$$

where **C**^*ϕ*^ is a covariance matrix. All solutions *ω*^*ϕ *^with *λ *≠ 0 lie in the space spanned by *ϕ*(**x**_1_),…, *ϕ*(**x**_*N*_) where N is the number of samples, and there exist coefficients *α*_*i *_such that

(13) $$\omega^{\phi }=\overset{N}{\underset{i=1}{\sum }}\alpha_{i}\cdot \phi (x_{i})$$

Denoting an *N *×* N *matrix K by

(14) $$K_{i,j}=K(x_{i},x_{j})=\phi (x_{i})\cdot \phi (x_{j})$$

the kernel PCA problem becomes [58]

(15) $$N\cdot \lambda \cdot K\cdot \alpha =K^{2}\cdot \alpha =N\cdot \lambda \cdot \alpha =K\cdot \alpha$$

where *α* denotes a column vector with entries *α*_1_,…,*α*_*N*_. The above derivation assumes that all the projected samples *ϕ*(**x**) are centered in $R^{f}$. In this work, we have used the Gaussian kernel PCA:

(16) $$e^{\frac{{∥x-y∥}^{2}}{2\cdot \sigma^{2}}}$$

We found two advantages of nonlinear kernel PCA: first, nonlinear principal components afforded better recognition rates and second, the performance for nonlinear components can be further improved by using more components than possible in the linear case [59].

## Appendix 2: Partial Least Squares SIMPLS algorithm

The SIMPLS algorithm [60] was proposed by Sijmen de Jong in 1993 as an alternative to the NIPALS algorithm for PLS. The main difference to NIPALS is the kind of deflation. In SIMPLS, no deflation of the centered data matrices **X** and **Y** is made, but the deflation is carried out for the covariance matrix, or more precisely, the cross-product matrix **S **=** X**^*T*^**Y **between the *x*-data and *y*-data [61]. SIMPLS algorithm can be described as follows: 

1. initialize **S**_0_ =** X**^*T*^**Y **and iterate steps 2 to 8 for *j *= 1,…,*n*

2. if *j *= 1, **S**_*j *_=** S**_0_ else, $S_{j}=S_{j-1}-P_{j-1}{\left(P_{j-1}^{T}P_{j-1}\right)}^{-1}P_{j-1}^{T}S_{j-1}$

3. compute **w**_*j *_as the first singular vector of **S**_*j*_

4. $w_{j}=\frac{w_{j}}{∥w_{j}∥}$

5. **t**_*j *_=** Xw**_*j*_

6. $t_{j}=\frac{t_{j}}{∥t_{j}∥}$

7. $p_{j}=X_{j}^{T}t_{j}$

8. **P**_*j *_= [**p**_1_,**p**_2_,…,**p**_*j*−1_]

The resulting weights **w**_*j *_and scores **t**_*j*_ are stored as columns in the matrix **W **and **T **respectively.

The nonlinear kernel PLS method is based on mapping the original input data into a high dimensional feature space [62]. SIMPLS needs to be reformulated into its kernel variant (in this work Gaussian kernel PLS pls LMNN transformation Acc result is shown in Table 2), assuming a zero mean nonlinear kernel PLS.

## Competing interests

The authors declare that they have no competing interests.

## Author’s contributions

The authors contributed to the selection and discussion of the literature reviewed in this work. The authors participated in the conception and preparation of the final manuscript. All authors read and approved the final manuscript.

## Pre-publication history

The pre-publication history for this paper can be accessed here:

http://www.biomedcentral.com/1472-6947/12/79/prepub
